# Isolation and cultivation of adult primary bovine hepatocytes from abattoir derived liver

**DOI:** 10.17179/excli2016-794

**Published:** 2016-12-22

**Authors:** Sonja Ehrhardt, Marion Schmicke

**Affiliations:** 1 University of Veterinary Medicine, Clinic for Cattle, Endocrinology, Bischofsholer Damm 15, 30173 Hannover, Germany

**Keywords:** hepatocyte, liver, cell culture, cattle, sandwich

## Abstract

The aim was to establish a cell culture of adult primary bovine hepatocytes obtained from liver following slaughter and to cultivate the cells in a sandwich culture. Cells and medium samples were taken after separation of cells (day 0), during monolayer (days 1, 2 and 3) and during sandwich culture (days 1, 2, 3, 4, 7, 10 and 14). The mRNA expression of *BAX*, *BCL2L*, *FAS*, *IGF-1* and *GHR1A* was measured as well as urea and LDH. Hepatocytes were obtained by using a two-step collagenase perfusion and were purified thereafter by density gradient centrifugation. The viability was 68.2 ± 9.5 %. In sandwich culture, cells have a typical polygonal hepatocyte-like shape, build cell-cell contacts, and show irregularity of cell borders suggesting bile canaliculi generation. The *BAX* mRNA expression increased on day 1 as well but decreased steadily until day 3 and remained constant for 14 days. Urea- and LDH-concentrations increased from day 4 to day 7. In conclusion, we found that it is possible to gather viable primary hepatocytes from adult bovine liver after slaughter, and that cells gathered this way show typical morphologies, urea-production and low LDH-leakage especially at day 4 in a sandwich system.

## Introduction

Studies addressing liver metabolism is crucial to the field of dairy science. However, the lack of suitable and well characterized models of adult primary bovine hepatocyte cell culture models limits the possibility for in vitro studies. Primary hepatocytes obtained from adult mice and rats are widely used for toxicological and metabolic studies and well standardized (Hewitt et al., 2007[5]; Shulman and Nahmias, 2013[12]). Currently, the majority of research on bovine hepatocytes is done with cells obtained from calves in monolayer culture (Donkin and Armentano, 1993[1]; Zhang et al., 2016[17]). For primary hepatocytes however sandwich culture is currently the most suitable method. In a sandwich culture, cells are cultivated between two layers of collagen (Godoy et al., 2013[4]). For studies using adult bovine hepatocytes, the recovery of vital hepatocytes from adult cow livers immediately after slaughter would be of interest in terms of reducing animal experiments. The process of obtaining vital hepatocytes from slaughterhouse-derived liver has already been described for equine (Stefanski et al., 2013[13]), pigs (Gerlach et al., 1993[2]) and recently for buffalo liver (Panda et al., 2015[9]). As mentioned above studies on bovine primary hepatocytes, by the majority obtain the hepatocytes from neonates or calves surgically and then euthanize the animals (Donkin and Armentano, 1993[1]; Li et al., 2013[7]; Zhang et al., 2016[17]). Donkin and Armentano (1993[1]) have described a process for the culturing of primary bovine hepatocytes up to 41 h after separation from male calves. Giantin et al. (2012[3]) described a method for isolating primary hepatocytes from the caudate lobe after slaughter in cattle. They have conducted viable cell-cultures from these cells for 24 h in a monolayer system. The present study aimed to establish a protocol for gathering viable primary hepatocytes from abattoir derived liver from adult cattle, and the culturing of these cells in a sandwich culture. Basic parameters should be tested (apoptosis [*BAX*, *BCL2L*, *FAS*], urea-production, LDH-leakage) to assess the vitality of cells in the sandwich culture and comparing these with a monolayer culture. Moreover, the expression of *GHR1A*, and *IGF-1* mRNA was of interest for possible further studies on GHR1A expression in dairy cows.

## Materials and Methods

### Liver perfusion technique

All chemicals were purchased from Sigma Aldrich, St. Louis, MO, USA, except where noted. The caudate lobe of the liver from adult bulls was collected a maximum of 30 min after slaughter (Westfleisch Minden-Lübbecke, Germany) and rinsed with 100 mL ice-cold EuroCollins buffer (Vinken et al., 2014[16]) by insertion of a buttoned cannula into major veins. After transportation of the liver in EuroCollins buffer on ice to the cell culture lab, the liver was rewarmed (5 min in 200 mL 20 C EuroCollins buffer), and four buttoned cannulas were inserted into vessels and fixed by tissue glue (Histoacryl, Braun Surgical S.a., Rubi, Spain). The prepared lobe was placed on a Buchner funnel, and a two-step perfusion using a peristaltic pump (Behr Labortechnik GmbH, Düsseldorf, Germany) was performed. All steps were conducted using sterile conditions under a cell culture hood. The perfusion buffer 1 was saturated with carbogen (95 % O_2_/5 %CO_2_) 30 min prior to the perfusion. 500 mL of perfusion buffer 1 (142 mM NaCl, 0.5 mM KCl, 9.2 mM HEPES, 1 mM EGTA, 0.005 mM glucose, 1.74 nmol/L bovine insulin, 100 nM dexamethasone, 0.2 µM Na-pyruvate (Biochrom, Berlin, Germany), pH 7.4) at 38.5° C was purged, followed by 200 mL perfusion buffer 2 in a non-circulating system (68 mM NaCl, 0.6 mM KCl, 9.2 mM HEPES, 4.8mM CaCl_2_), and, finally, 144 IU collagenase P (90 mg, Roche, Indianapolis, IN, USA; dissolved in perfusion buffer 2) was pumped throughout the liver lobe using a re-circulating system. The timing of collagenase perfusion was stopped when the tissue irreversibly lost its structure (between 6-10 min). After the two-step perfusion, the perfused lobe was transferred into a sterile glass petri dish, and the glissonian capsule was disrupted with a sterile scalpel. Hepatocytes were collected into PBS containing 20 % fetal bovine serum (FBS, Pan BioTech, Aidenbach, Germany). The cell solution was then filtered through gauze, centrifuged (80 g for 3 min at 4° C), and washed with ice-cold PBS. The pellet was then suspended in a defined volume of Williams E Medium with stable Glutamine and without Phenol red (PAN BioTech) and centrifuged again in order to wash the cells. Hepatocytes were purified using Easycoll (Millipore, Massachusetts, USA; 1.124 g/mL, 1000g, 4° C, in a 15 ml Falcon Tube). Cells were counted, and viability was determined by a trypan blue exclusion test, conducted by mixing 1 part cell solution and 5 parts trypan blue. 

### Cell culture

The cells were seeded on six-well plates that were previously coated with rat tail collagen (1 mg/mL Collagen, Roche) and submerged in 2 mL Williams E Medium (containing 10 % FBS, 1.74 nmol/L bovine insulin, 100nM dexamethasone, 0.2 µM Na-pyruvate (Biochrom, Berlin, Germany), 100 U/mL penicillin, 0.1 mg/mL streptomycin, 10 µg/mL gentamycin) at a density of 1 x 10^6^ cells/well (1.37 x 10^5^ cells/cm^2^). After 3 h seeding, the medium was removed, and a second layer of rat tail collagen (pH 7.4 after dissolving in an acidified solution) was added to the cells to maintain the sandwich culture. William's E Medium without FBS (2mL) was added after the second layer of collagen was solidified. Cells were cultured in a humidified atmosphere at 37° C and 5 % CO_2_. The media was changed 24 h after separation to a hormone-defined medium that was equivalent to the Williams E Medium described above, but without FBS. The cells were monitored for three days in monolayer and 14 days in sandwich culture. The viability during the cell culture was tested qualitatively on day 1, 7, 10 and 14 by using Live/Dead Viability/Cytotoxicity Kit (Life technologies, Carlsbad, CA, USA) and was defined as good if > 95 % of all cells per visual field were stained green indicating living cells. To analyze the mRNA expression of apoptosis genes, *GHR1A* and *IGF-1*, cell samples were collected during monolayer and sandwich culture from 7 livers in technical duplicates as mentioned above and samples immediately frozen in liquid nitrogen (after EasyColl (= day 0), monolayer: day1,2 and 3; sandwich: day 1, 2, 3, 4, 7, 10 and 14). Thereafter, cells were kept at -80° C until analysis. 

### Analysis of mRNA-expression

The relative abundance of *BAX*, *BCL2L*, *FAS*, *GHR1A*, and *IGF-1* mRNA in the cells samples was detected using RT-qPCR. Total RNA was extracted by appending 1 mL Trizol reagent to the cell samples (pooled from 2 wells). After adding 0.2 mL chloroform and centrifuging, the mixture separates into 3 phases with the upper clear aqueous phase containing the RNA. The aqueous layer was taken and mixed with 0.5 mL isopropanol to precipitate the RNA. Thereafter the mRNA was washed with 1 mL ethanol and diluted in 25 µl RNase free Water. To assess the extracted RNA quality and quantity, relative integrity and to exclude contamination with DNA, an RNA 6000 Nano Assay Kit using the Agilent 2100 Bioanalyzer (Agilent Technologies, Böblingen, Germany) was used. To transcribe RNA into cDNA, the BioRad Real Time System CFX96 1000 Touch (BioRad, Munich, Germany) was used according to the manufacturer's instructions and as previously described (Piechotta et al., 2013[10]). A PCR reaction mix containing 5 ng/µL cDNA, 10 μL of MESA GREEN PCR MasterMix Plus for SYBR Assay (Eurogentec, Cologne, Germany), and 0.2 μM of each primer (Eurofins MWG Operon, Ebersberg, Germany) for the genes of interest was used. The primers were either previously described (Piechotta et al., 2013[10]) or constructed in April 2015 using http://biotools.umassmed.edu/cbioapps/primer3_www.cgi (Table 1(Tab. 1)). The PCR cycler was programmed as follows: RNA denaturation at 95° C for 15 min followed by 43 cycles of 95° C for 15 s, 60° C for 30 s and 72° C for 30 s for amplification. SYBR Green was used to visualize the fragments. A melting curve was generated to verify PCR fragments. This was initiated at 55° C, increasing to a final temperature of 95° C, with a temperature increase of 0.5° C every 10 s. Finally, the relative abundance of *BAX*, *BCL2L*, *FAS*, *GHR1A* and *IGF-1* mRNA in the cell samples, relative to the housekeeping genes *ß-actin* and *GAPDH*, was calculated using Microsoft Excel 2010 (Microsoft Corporation, Redmond, WA, USA). The ΔCt method was used to calculate the relative abundance of mRNA in the cell samples. The mean value of the Ct-merit of a specific gene was subtracted from the mean value of the two housekeeping genes (*ß-actin* and *GAPDH*) and calculated by the formula 2-ΔCt x 100.

### Urea- and LDH-measurement

The Urea-concentration was measured by using the Urea Assay Kit ab83362 (abcam, Cambridge, UK) according to the manufacturer's instructions, and the LDH concentration was measured by using the Lactate Dehydrogenase Activity Assay Kit (Sigma-Aldrich, St. Louis, USA). 

### Statistical analyses

Statistical analyses were carried out using Graph Prid Prism (GraphPad Software, Inc., Ver 6). The mRNA data was tested for normal distribution using the Shapiro-Wilk test and differences between days were analyzed by a Wilcoxon-signed-rank test. Differences between monolayer and sandwich were tested by using a non-parametric Mann-Whitney* U* test. A P value of < 0.05 was considered significant. Data is presented as mean ± SD.

## Results

### Cell morphology and culture

**Figure 1:**
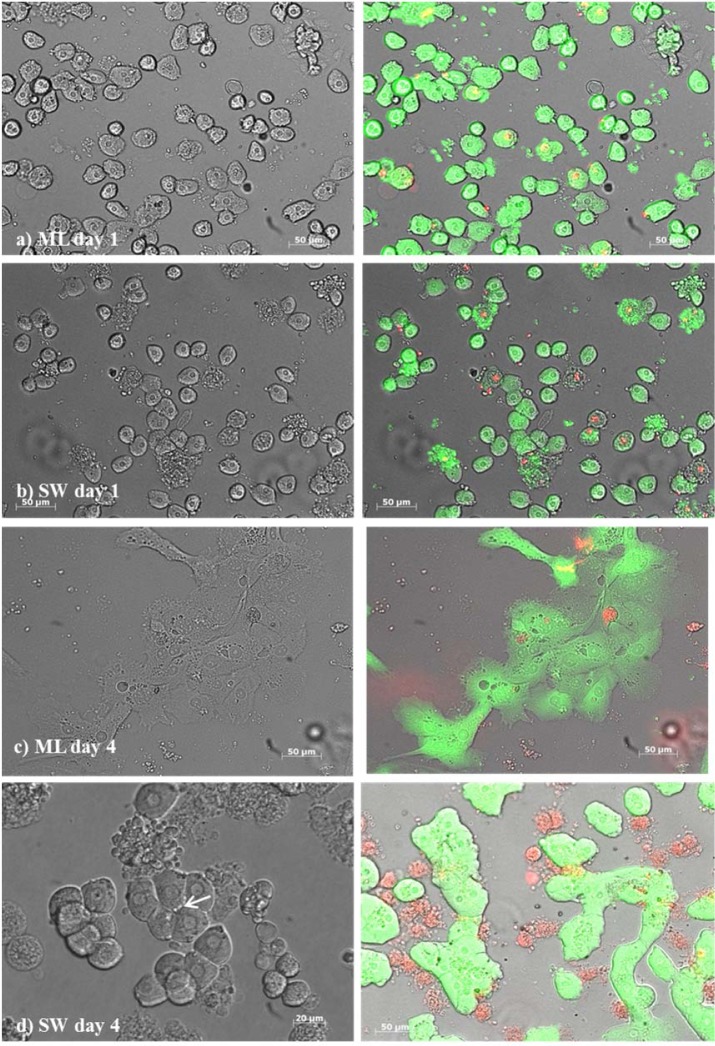
Primary bovine hepatocytes from liver lobes after slaughtering in a monolayer (ML) culture [a) day 1, c) day 4)] and in a sandwich culture (SW) [b) day 1, d) day 4] between two layers of collagen. The arrow in picture d) indicates an irregular cell border suggesting bile canaliculi formation. Respectively, live cells indicated by green and dead cells by red color using the LIVE/DEAD™ assays by ThermoFischer Scientific in the monolayer and sandwich culture. Green cells with red colored nucleus indicate apoptotic cells.

During the establishment of the protocol 20 liver lobes were perfused and from 13 perfused liver lobes, viable cells were collected successfully. The number of these viable cells derived from the established protocol ranged from 2040000 to 8310000 (5064038 ± 1961 320 cells; ~ 5 x 10^4^ cells /g liver). The viability of cells following Easycoll separation was 68.2 ± 9.5. Due to organizational reasons cells from seven livers were used for the subsequent monolayer and sandwich cultures. After separation, the hepatocytes displayed a roundish, spherical shape (Figures 1a, b(Fig. 1)). After adhesion, the morphology had changed to a polygonal hepatocyte-like cell shape (Figure 1d(Fig. 1)). During the sandwich culture, the cells built cell-cell contacts and show irregularity of cell borders suggesting canaliculi formation (Figure 1d(Fig. 1)), a nucleus or two nuclei per cell were present for four days, whereas in monolayer culture, the cytoplasm became granulated after one day and the cells showed fibrocyte-like structure after two days (Figure 1c(Fig. 1)).

### Gene expression

**Figure 2:**
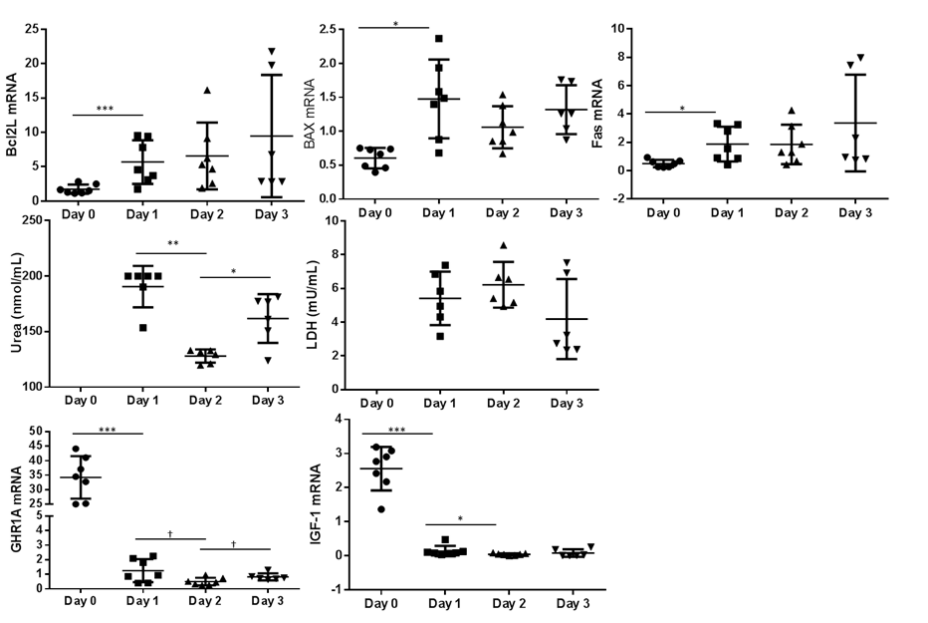
Monolayer culture: Relative abundance of mRNA expression of apoptosis markers (*BCL2L*, *BAX* and *FAS*) as well as *GHR1A* and *IGF-1* from day 0 (cells after Easycoll isolation) and day 1, 2 and 3. The urea and LDH concentration was determined in the cell culture medium supernatant but not after purification of cells (day 0). Significant differences were indicated as follows: * P < 0.05, ** P < 0.01, *** P < 0.001 . *BCL2L *= B-cell lymphoma 2 like 1, BAX = BCl2 associated X, *FAS *= Fas (CD95L), LDH = Lactate dehydrogenase, *GHR1A *= Growth hormone receptor 1A transcript, *IGF-1 *= Insulin-like Growth Factor-1

**Figure 3:**
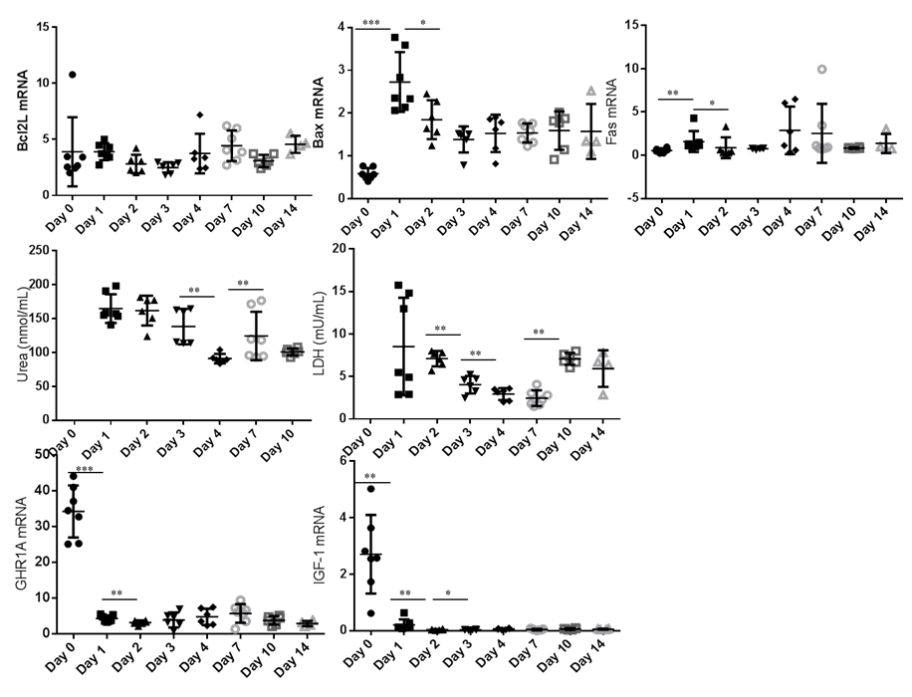
Sandwich culture: Relative abundance of mRNA expression of apoptosis markers (*BCL2L*, *BAX* and *FAS*) as well as GHR1A and IGF-1 from day 0 to day 14 (cells after Easycoll isolation). The urea and LDH concentration was determined in the cell culture medium supernatant but not after purification of cells (day 0). Significant differences were indicated as follows: * P < 0.05, ** P < 0.01, *** P < 0.001. *BCL2L *= B-cell lymphoma 2 like 1, BAX = BCl2 associated X, *FAS *= Fas (CD95L), LDH = Lactate dehydrogenase, *GHR1A *= Growth hormone receptor 1A transcript, *IGF-1 *= Insulin-like Growth Factor-1

In the monolayer culturing system the expression of the pro apoptotic markers *BAX* and *FAS* increased between day 0 and day 1 (P < 0.05) (Figure 2(Fig. 2)). During 14 days of culture of hepatocytes between two layers of collagen, the expression of apoptotic markers remains constant (Figure 3(Fig. 3)). While comparing both culturing systems *BAX* expression was higher in sandwich culture during the first 2 days (Table 2(Tab. 2)). The *GHR1A* and *IGF-1* expression significantly decreased in both culturing systems between day 0 and day 1, and further from day 1 to day 2 (Figures 2(Fig. 2) and 3(Fig. 3)). Interestingly, the *GHR1A* expression was significantly higher during the sandwich culture for three days if compared to the monolayer system (Table 2(Tab. 2)). 

### Urea- and LDH-concentration

The urea concentration sharply decreased between day 1 and 2 in the monolayer system (Figure 2(Fig. 2)). In the sandwich culture however the urea production remains constant for the first three days and decreased to day 4 but increased again between day 4 and 7 (Figure 3(Fig. 3)). For the first three days the urea production is almost comparable between monolayer and sandwich culturing condition but was slightly higher in the sandwich culture on day two compared to the monolayer (Table 2(Tab. 2)). The LDH concentration in the medium supernatant was comparable between the first three days in the monolayer system and decreased in the sandwich culture to day 3 and increased again between day 7 and 10 (Figure 3(Fig. 3)). During the first three days the LDH concentration was comparable between the medium supernatants obtained from monolayer and sandwich culture (Table 2(Tab. 2)). 

## Discussion

In our study, an average of 68 % viable hepatocytes was achieved. This result was slightly lower compared to Giantin et al. (2012[3]) who achieved 88 % viable cells and who also obtained liver cells from two heifers after slaughter or to Panda et al. (2015[9]) who obtained 82 % viable cells from buffalo liver after slaughter. It is known from rat hepatocytes that after 72 h of culture on collagen monolayer, the cells develop perturbed cell morphology, and cells spread out and form fibroblast-like protrusions. These results have also been confirmed after 48 h in monolayer cultures of bovine hepatocytes (Tuschl et al., 2009[14]; Tuschl and Mueller, 2006[15]) and in the present study. In contrast to Giantin et al. (2012[3]), in the present study, bovine hepatocytes were cultured after isolation in a sandwich culture system. The polygonal cell shape of the cells was still stable after 14 days, and this result is comparable to results obtained from rat hepatocytes cultured between two layers of collagen (Tuschl and Mueller, 2006[15]). Moreover, the cells showed irregularity of cell borders indicating bile canaliculi formation (LeCluyse et al., 1994[6]). However, in future studies the bile canaliculi formation and function should be proven e.g. by bile canalicular excretion kinetics of CMFDA (5-chloromethylfluorescein diacetate) (Reif et al., 2015[11]).

The apoptotic signals *BAX* and *FAS* increased in the sandwich culture between day 0 and the first day in culture in the present study but decrease thereafter steadily; indicating low long term apoptosis of cells. It is unclear why hepatocytes initially showed higher *BAX* expression in sandwich than in monolayer culture. One reason could be that already detached non-viable cells were removed from the monolayer due to medium chance but remained in the sandwich culture due to the collagen overlayer. Lactate dehydrogenase is used to detect necrotic cell death caused a disruption of the cell membrane and the release of the cytoplasmatic enzyme LDH (Maes et al., 2015[8]) and urea production as marker for cell metabolism and liver specific function. As metabolic marker albumin has to be measured in future studies to further characterize the primary bovine hepatocytes. Urea concentration was constant for three days in the sandwich culture and higher on day 2 compared to cells cultivated in a monolayer. Together with lower LDH concentrations in the medium supernatant in the sandwich culture these results indicate better hepatocyte viability in the sandwich culture. 

We showed that both *GHR1A* and *IGF-1* mRNA expression significantly decrease between day 0 and day 1 in both culturing systems but interestingly, the *GHR1A *mRNA expression was significantly higher during the first three days in sandwich culture compared to the monolayer culturing system. However, further studies are necessary first to optimized the culturing conditions to eventually preserve the *GHR1A* and *IGF-1* expression and to test the functionality of the GHR in vitro by adding growth hormone. 

In conclusion, viable hepatocytes can be gathered from abattoir derived liver and cultured in sandwich-culture. Typical hepatocyte like morphology was observed for 14 days in sandwich culture. Based on the morphological shape, low expression apoptotic markers, urea production and low LDH leakage bovine hepatocytes show the highest vitality on day 4 in sandwich culture. This technique provides the basis for different studies with adult bovine primary hepatocytes. 

## Acknowledgements

We thank J. Hengstler and R. Stöber (Leibniz Research Centre for Working Environment and Human Factors, Dortmund) and G. Damm (Charité Berlin) for practical training with primary hepatocytes, N. Hambruch for support with microscopy as well as Dr. Dusinski for kindly providing livers from the slaughterhouse Minden (Westfleisch GmbH). 

## Conflict of interest

There is no conflict of interest.

## Figures and Tables

**Table 1:**
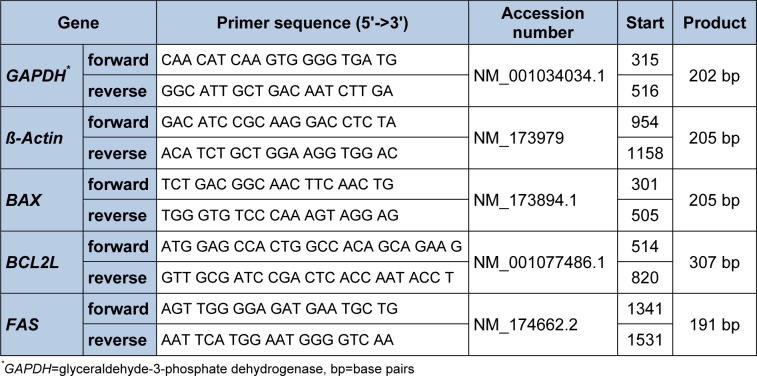
Quantitative real-time PCR primers used for the genes of interest in samples obtained after separation of cells and during monolayer and sandwich culture of primary bovine hepatocytes

**Table 2:**
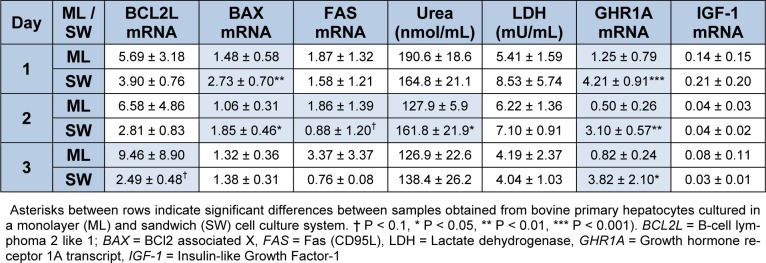
Comparison of mRNA Expression of apoptosis markers, *GHR1A* and *IGF-1* as well as urea and LDH medium concentration between primary bovine hepatocytes obtained after slaughtering between monolayer (ML) and sandwich culture (SW) on day 4 after separation of cells, n=6 repetitions each). Data is presented as mean ± SD.
